# Resveratrol Enhances Airway Surface Liquid Depth in Sinonasal Epithelium by Increasing Cystic Fibrosis Transmembrane Conductance Regulator Open Probability

**DOI:** 10.1371/journal.pone.0081589

**Published:** 2013-11-25

**Authors:** Shaoyan Zhang, Angela C. Blount, Carmel M. McNicholas, Daniel F. Skinner, Michael Chestnut, John C. Kappes, Eric J. Sorscher, Bradford A. Woodworth

**Affiliations:** 1 Department of Surgery/Division of Otolaryngology, University of Alabama at Birmingham, Birmingham, Alabama, United States of America; 2 Department of Cell, Developmental and Integrative Biology, University of Alabama at Birmingham, Birmingham, Alabama, United States of America; 3 Gregory Fleming James Cystic Fibrosis Research Center, University of Alabama at Birmingham, Birmingham, Alabama, United States of America; 4 Department of Medicine, University of Alabama at Birmingham, Birmingham, Alabama, United States of America; Hospital of the University of Pennsylvania, United States of America

## Abstract

**Background:**

Chronic rhinosinusitis engenders enormous morbidity in the general population, and is often refractory to medical intervention. Compounds that augment mucociliary clearance in airway epithelia represent a novel treatment strategy for diseases of mucus stasis. A dominant fluid and electrolyte secretory pathway in the nasal airways is governed by the cystic fibrosis transmembrane conductance regulator (CFTR). The objectives of the present study were to test resveratrol, a strong potentiator of CFTR channel open probability, in preparation for a clinical trial of mucociliary activators in human sinus disease.

**Methods:**

Primary sinonasal epithelial cells, immortalized bronchoepithelial cells (wild type and F508del CFTR), and HEK293 cells expressing exogenous human CFTR were investigated by Ussing chamber as well as patch clamp technique under non-phosphorylating conditions. Effects on airway surface liquid depth were measured using confocal laser scanning microscopy. Impact on CFTR gene expression was measured by quantitative reverse transcriptase polymerase chain reaction.

**Results:**

Resveratrol is a robust CFTR channel potentiator in numerous mammalian species. The compound also activated temperature corrected F508del CFTR and enhanced CFTR-dependent chloride secretion in human sinus epithelium *ex* *vivo* to an extent comparable to the recently approved CFTR potentiator, ivacaftor. Using inside out patches from apical membranes of murine cells, resveratrol stimulated an ~8 picosiemens chloride channel consistent with CFTR. This observation was confirmed in HEK293 cells expressing exogenous CFTR. Treatment of sinonasal epithelium resulted in a significant increase in airway surface liquid depth (in µm: 8.08+/-1.68 vs. 6.11+/-0.47,control,p<0.05). There was no increase CFTR mRNA.

**Conclusion:**

Resveratrol is a potent chloride secretagogue from the mucosal surface of sinonasal epithelium, and hydrates airway surface liquid by increasing CFTR channel open probability. The foundation for a clinical trial utilizing resveratrol as a therapeutic intervention to increase mucociliary transport and airway surface liquid hydration in sinus disease is strongly supported by these findings.

## Introduction

Chronic rhinosinusitis (CRS) affects 16% of the US population with an estimated aggregated cost of 8.6 billion dollars annually in healthcare expenditures[1–3]. CRS causes significant decrements in patient quality of life in terms of nasal airway specific morbidity, as well as general health and vitality. Afflicted patients demonstrate decreased scores for physical pain and social function compared to those suffering from chronic obstructive pulmonary disease, congestive heart failure or angina[2]. Conventional CRS interventions have been limited by bacterial resistance incurred with antibiotic overuse and the deleterious side effects of steroids. Safe, but effective, compounds that enhance Cl^-^ transport could provide significant therapeutic advantages in this regard.

Mucociliary clearance (MCC) is vital to maintaining healthy sinus mucosa. Impairment of MCC predisposes to chronic infections of the upper respiratory tract which confer chronic airway inflammation and persistent changes in mucus transport[4,5]. The mucociliary apparatus is composed of multiple elements essential to function, one of the most important being airway surface liquid (ASL). The viscosity and depth of the ASL are determined in part by the balance of transepithelial transport of ions, including chloride (Cl^-^) and sodium (Na^+^). The cystic fibrosis transmembrane conductance regulator (CFTR) mediates the transport of a significant amount of Cl^-^ and HCO3^-^ in both the upper and lower respiratory epithelium. Disrupted Cl^-^ transport leads to dehydrated ASL and mucus stasis, most notably in the airway manifestations of cystic fibrosis (CF). Impaired MCC leads to chronic rhinosinusitis (CRS) and is insidious among the CF population. Ivacaftor (Vertex pharmaceuticals, Cambridge, Ma), the first approved compound of its kind to stimulate MCC by augmenting CFTR activity leads to dramatic improvement in CF related abnormalities of MCC in both upper and lower airways[6]. Here we provide evidence that therapeutic strategies aimed at improving MCC among non CF individuals with CRS could represent a new and powerful approach to therapy for this very prevalent disease. 

In this context, flavonoids and related natural products comprise a group of plant molecules reported to have Cl^-^ secretagogue activity[7]. Agents such as these also exhibit anti-inflammatory and other useful properties[8,9]. Resveratrol is an organic polyphenol from a family structurally related to the flavonoids, and found in many plants and vegetables, including grapes[10]. Widely recognized for its immune-modulator activity (attributable to inhibition of NF-κB)[11], resveratrol markedly dampens inflammatory pathways mediated by inducible nitric oxide synthase (iNOS), COX-2, granulocyte-macrophage colony-stimulating factor (GM-CSF), and IL-8 (or the murine homologue KC)[11]. We reported previously that resveratrol can stimulate transepithelial Cl^-^ secretion and may therefore be suitable for development as a topical therapeutic for sinonasal disease[12]. The objectives of the current study were to investigate the mechanism of resveratrol on CFTR channel function and expression, open channel probability ( Po), mucociliary activity, and hydration of airway surface liquid, endpoints predictive of beneficial effects *in vivo*.

## Methods

University of Alabama at Birmingham Institutional Animal Care and Use Committee and Institutional Review Board approval were obtained prior to initiation of the study. Written informed consent was obtained from each participant on a document approved by the Institutional Review Board. 

### Tissue Culture

Normal sinonasal mucosa was obtained intraoperatively from patients undergoing endoscopic surgery for pituitary tumor, benign sinonasal tumor or cerebrospinal fluid leak repair and investigated by Ussing chamber analysis or establishment of primary cell culture. Primary sinonasal epithelial cells from humans, mice, and pigs, as well as CFBE immortalized cell lines expressing either wild type or F508del CFTR were cultured at an air-liquid interface according to previously established protocols[5,13-18] and used to evaluate whether resveratrol dependent CFTR activation is conserved across species. Murine nasal septal epithelial (MNSE) cells and a recombinant CFTR-expressing HEK293 cell line (“D060”) were employed to investigate effects of resveratrol on open probability (Po) of single CFTR ion channels. Methods for membrane patch analysis of CFTR in primary cells, including single channel characteristics of the native protein, have not been reported previously, and utilized protocol adjustments described below. All MNSE cells were obtained from congenic C57/BL6 wild type and CFTR^-/-^ mice. Primary nasal epithelial cells were prepared and cultured[15] on collagen coated Costar 6.5-mm-diameter permeable filter supports (Corning, Lowell, MA) submerged in culture media. 

#### HEK293 cells expressing native human CFTR

Briefly, 293F cells (Invitrogen) were transduced with a lentiviral vector (designated K2801) that constitutively expressed the rtTA2^S^-M2 transactivator[19] and a blasticidin resistance gene under control of an EF1α promoter. Transduced cells were selected with blastocidin (10 µg/ml). Single cell derived clonal cultures were generated and screened for responsiveness by infection with a lentiviral vector comprising TRE-EGFP. A clonal culture (designated 293F.M2 (or D044)) exhibiting favorable characteristics was selected for subsequent studies. D044 cells were transduced with a VSV-G pseudotyped packaged lentiviral vector (designated K2933) comprising the native human CFTR gene downstream of the tet-responsive element (TRE), an internal ribosomal entry site (IRES), and a fused (in-frame) puromycin-T2A-EGFP open reading frame (TRE-CFTR-IRES-puro.T2A.GFP), wherein T2A comprises the peptide sequence EGRGSLLTCGDVEENPGP[20]. Transduced cells (designated HEK293-CFTR or D060) were enriched by selection in medium containing puromycin (5 µg/ml) subsequent to induction with doxycycline (1 µg/ml). Doxycycline induced expression of CFTR protein in the D060 cell line utilized methods described previously[21]. 

### Electrophysiology

#### Short Circuit (I_SC_) Measurements

Tissue specimens were immediately stored in an ice cold DMEM/F-12 media (Invitrogen, USA) following surgical harvest and transferred to the laboratory within 15 minutes. A thin layer of epithelial tissue was dissected from the surgical specimen, placed on a slider with an open area of 0.031 - 0.71cm^2^, and mounted between Ussing-type hemichambers (Easy Mount, Physiologic Instruments Inc. CA. USA). Transwell inserts (Costar) containing primary or immortalized monolayers were configured in Ussing chambers (VCC 600; Physiologic Instruments Inc. CA. USA) in order to investigate pharmacologic manipulation of vectorial ion transport. Tissues or cell monolayers were continuously analyzed under short circuit conditions following fluid resistance compensation using automatic voltage clamps. Batch solutions for the transwell filters were warmed to 37°C, and each solution continuously gas lifted with 95%O_2_-5%CO_2_. Drugs included amiloride (100 µM) to block sodium transport, resveratrol (100 µM), ivacaftor (10 µM), and CFTR(inh)172 (10 µM) to inhibit CFTR-mediated I_SC_. Corresponding DMSO (vehicle) control solutions for resveratrol and ivacaftor were studied in parallel. The I_SC_ was assessed at one current measurement per second. By convention, a positive deflection in I_SC_ was defined as the net movement of anions in the serosal to mucosal direction.

#### Single Channel Studies

Conventional patch clamp techniques were adapted to record single channel currents by cell-attached and inside-out configuration using MNSE and HEK293 cells expressing WT CFTR. Recording pipettes were constructed from borosilicate glass capillaries (Warner Instruments, Hamden CT) using a Narishige PP83 microelectrode puller and fire polished with a PP90 microforge (Narishige, Tokyo). The pipettes were partially filled with a solution containing (in mmol/l) 150 CsCl, 1 CaCl_2_, 1 MgCl_2_ and 10 HEPES (pH 7.2) and had tip resistances of 6-8 MΩ. Experiments were performed at room temperature (20-22°C). Single channel currents were obtained using an Axopatch 200B patch clamp amplifier (Axon Instruments (AI), Molecular Devices, USA) with voltage commands and data acquisition controlled by Clampex software (pClamp 10, Axon Instruments) and digitized (Digidata 1440A interface, AI) at a sampling frequency of 1 kHz. HEK293 cells seeded on glass coverslips or MNSE cells on filters mounted in a flow through chamber To obtain seals, bath solutions contained (in mmol/l) 140 NaCl, 4.0 KCl, 1.8 CaCl_2_, 1.0 MgCl_2_, 10 glucose, 10 HEPES, pH 7.4. For inside out patches, bath solution contained (in mmol/l) 150 CsCl, 5 EDTA and 10 HEPES pH 7.4 designed to minimize channel rundown. Based on the dose-response relationship of (trans) resveratrol (Sigma, St. Louis, Mo.) and CFTR activation[12], the working concentration was 100 µM, a drug concentration routinely achievable in topically applied, superperfused, or aerosolized solutions in human subjects *in vivo*[22-24]. Vehicle controls were included in all studies [DMSO (0.02%)]. Confirmation that stimulated currents were due to CFTR activity was achieved using the inhibitor CFTR(inh)-172 at 10 µM. Single channel recordings were analyzed using pClamp 10 software (AI). Vcom = command potential. Tracings were filtered post acquisition at 500 Hz.

### Airway Surface Liquid Depth

Cells were washed 3 times with PBS followed by administration of 100 μM CellTracker^TM^ Green BODIPY**^®^** (Invitrogen, Cat#: C2102) to the basolateral medium and 10mg/ml Texas Red^®^ (Invitrogen, Cat #: D-1863) in FC-70 (Flourinert FC-70, Fisher, Cat #: NC 9062226) to the apical side. Resveratrol, resveratrol + CFTR(inh)-172 (10 µM) or vehicle (DMSO) control were added to apical (in 30 µl volume) chambers for 30 minutes and imaging performed with a Zeiss LSM 710 Confocal Microscope (20x objective). 

### Gene Expression

Total RNA was isolated with the RNeasy mini kit (Qiagen) according to manufacturer instructions. To prevent DNA contamination, samples were pretreated with RNase-free DNase (Qiagen) and column purified. The one-step Applied Biosystems PCR protocol was used to quantify CFTR transcripts with the *ABI* Prism 7500 sequence detection system and six serial dilutions of RNA isolates (Applied Biosystems, Foster City, CA). TaqMan OneStep PCR Master Mix Reagents Kit (ABI) was used for reverse transcription and PCR. Primers for murine CFTR, human CFTR and 18S rRNA were purchased from Assays on Demand (ABI); with assay ID for murine CFTR, Mm00445197_m1; and human CFTR, Hs00357011_m1. The thermocycler conditions were as follows: Stage 1: 48°C for 30 min; Stage 2: 95°C for 10 min; Stage 3: 95°C for 15 sec; Stage 4: 60°C for 1 min; 40 cycles. All CFTR values were normalized to 18S rRNA (from the same sample) according to the Applied Biosystems relative quantification method. Experiments were performed in triplicate. 

### Statistical analysis

Statistical analyses were conducted using GraphPad Prism 6.0 software (La Jolla, Ca) with significance set at P < 0.05. Statistical evaluation utilized paired and unpaired Student t tests, the Mann-Whitney rank sum test, or the analysis of variance followed by Tukey-Kramer multiple comparison test as appropriate. 

## Results

### Resveratrol activation of CFTR-mediated Cl^-^ transport is conserved across species

**Figure 1:**
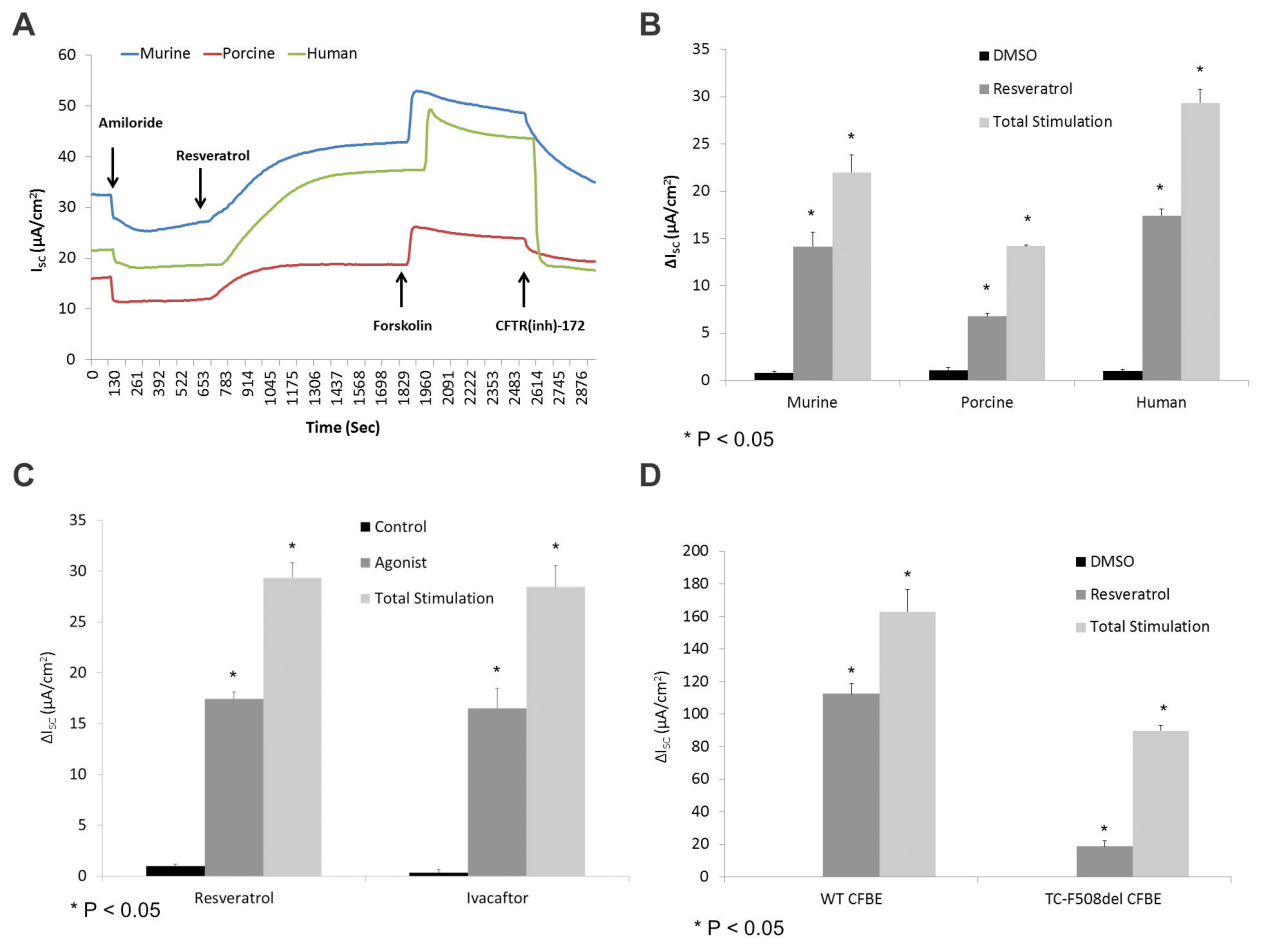
Resveratrol is a robust activator of CFTR-mediated Cl^-^ secretion in primary sinonasal epithelial cell cultures from several mammalian species and immortalized CFBE cells. (A) Representative Ussing chamber tracings demonstrate pharmacologic manipulation of ion transport showing resveratrol activation of Cl^-^ transport in murine, human, and porcine sinonasal epithelial cell cultures. By convention, a positive deflection in the tracing (ΔI_SC_) represents movement of anion in the serosal to mucosal direction. (B) Graphic representation of resveratrol (100 µM) stimulation vs. total stimulation [resveratrol (100 µM) + forskolin (20 uM)] for transepithelial Cl^-^ conductance in murine, human, and porcine primary nasal epithelial cultures. Significant stimulation (p<0.05) of Cl^-^ transport was demonstrated in primary sinonasal cultures as compared to untreated vehicle controls in all species. Total stimulation (addition of 20 µM forskolin) was also significantly greater than resveratrol alone (p<0.05). Resveratrol (as a percentage of total stimulation) consistently activated between 50 to 70% of total CFTR-mediated anion transport. (C) When compared to ivacaftor (10 µM) in human sinonasal epithelial cultures, resveratrol activates wild type CFTR-mediated anion transport to a similar degree. (D) Stimulation of wild type (WT) and temperature corrected (TC)-F508del CFTR was noted in the CFBE cell line.

The change in short-circuit current (ΔI_SC_ (µA/cm^2^)) attributable to CFTR activation by resveratrol was significantly increased across primary upper airway epithelia derived from several mammalian species (Figure 1A and 1B). Murine [(ΔI_SC_, 14.2+/-1.5 vs. 0.8+/-0.2(control)], human [(17.4 +/- 0.7 vs. 1.0 +/-0.2(control)], and porcine [(6.8 +/- 0.3 vs. 1.1 +/-0.3(control)] sinonasal airway epithelial cells in primary cultures exhibited significant stimulation (p<0.05) of CFTR-mediated Cl- secretion (Figure 1). Resveratrol activated between 50 and 70% of total CFTR-dependent I_SC_ as judged by addition of saturating (20 μM) forskolin (murine 22.0+/-1.8, human 29.4+/-1.5, and porcine 14.2 +/- 0.2). Resveratrol led to equivalent levels of CFTR-mediated anion transport when compared to ivacaftor (16.5+/-1.9 at an optimal concentration of 10 µM) in human sinonasal epithelial cultures (Figure 1C). Activation was also robust in a CFBE cell line expressing high levels of recombinant wild type CFTR [(112.5 +/- 6.2 vs. 0.2 +/-0.3(control), p<0.05)] and F508del CFTR following low temperature incubation at 25° C for 48 hours [(18.8 +/- 3.5 vs. 01+/- 0.1(control), p<0.05] indicating functionality with the “corrected” F508del protein (Figure 1D). Stimulation of low temperature incubated F508del CFTR was similar in magnitude to ivacaftor (20.4+/-2.4) (data not shown). 

### Resveratrol activates human sinonasal CFTR-mediated Cl^-^ transport ex vivo

**Figure 2:**
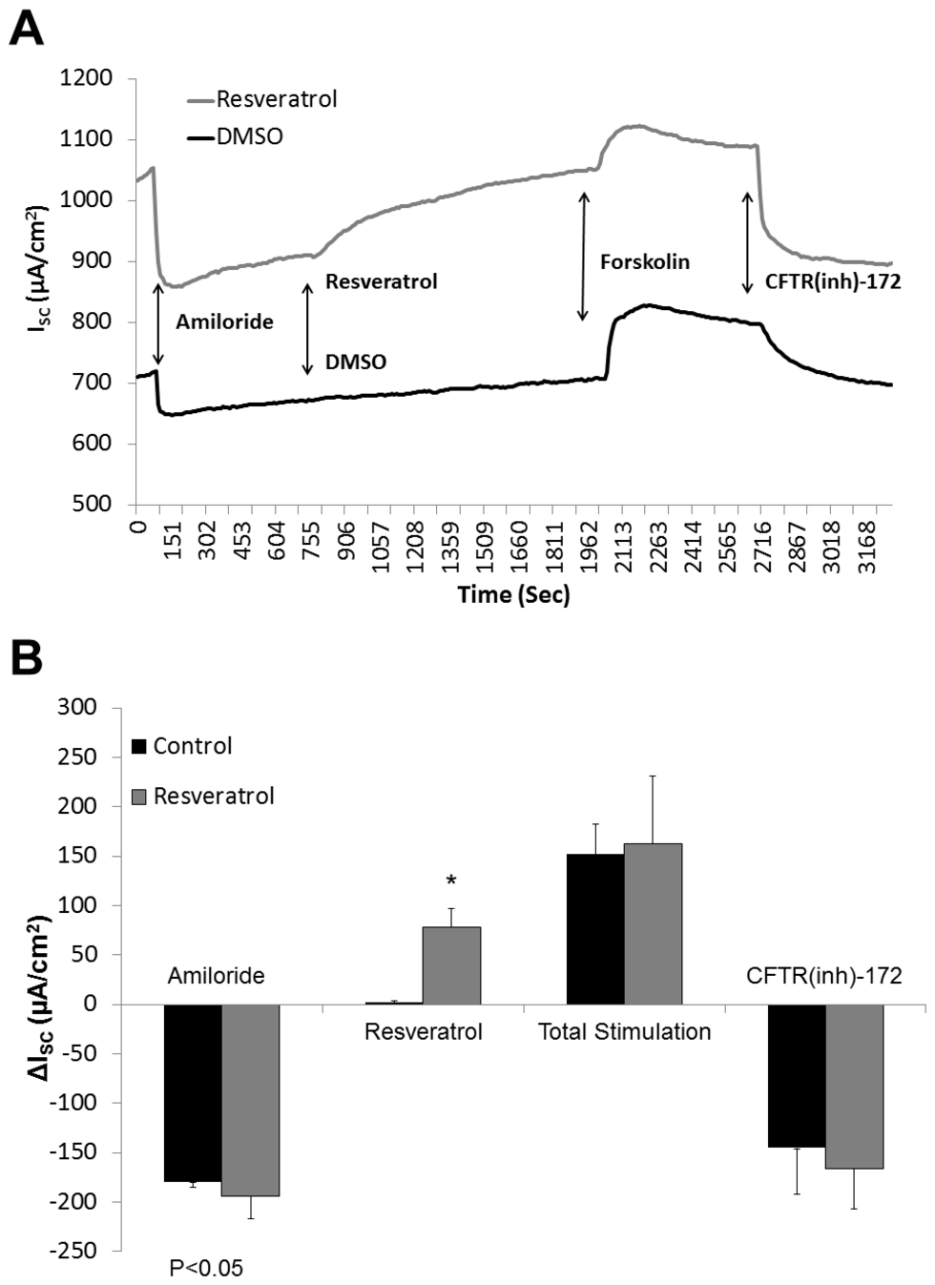
Resveratrol activates CFTR-mediated Cl^-^ secretion in human sinonasal mucosa *ex* *vivo*. Representative Ussing chamber tracings of full thickness human sinonasal tissue explants demonstrating robust activation of CFTR-dependent Cl^-^ secretion with 100 µM resveratrol (A). Ussing chamber summary data evaluating paired sinus mucosal samples from 5 individuals, and demonstrating significant activation (p<0.05) of *ex* *vivo* CFTR-dependent Cl^-^ transport (B).

Human ion transport activity was also evaluated in freshly excised sinus tissue from individuals undergoing endoscopic sinus surgery (n=5; paired samples) (Figure 2). Resveratrol robustly stimulated CFTR-mediated Cl^-^ secretion in human sinus explants [78.42 +/- 1.75 vs. 1.75 +/- 1.5 (control), p<0.05)]. Other parameters including amiloride-sensitive I_SC_ (-193.5 +/- 23.5 vs. 179.6 +/- 5.7 (no resveratrol)) representing epithelial sodium channel activity, total stimulated I_SC_ with forskolin (resveratrol + forskolin 163.0 +/- 67.9 vs. control + forskolin 152.4 +/- 30.0), and CFTR inhibition with CFTR(inh)172 (-165.7 +/- 40.9 vs. 145.2 +/- 46.8) were similar between groups, indicating that physiologic ion transport was intact among resveratrol treated and untreated mucosal samples. 

### Basal activity of CFTR channel in MNSE

**Figure 3:**
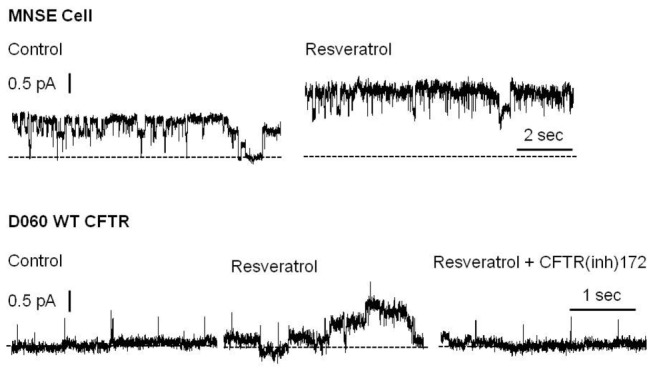
CFTR channel activity in MNSE cells. Constitutively active CFTR channel currents were recorded from a cell attached patch from MNSE. Two discernable channels were observed. Dotted line represents closed state and numbers to the left indicate holding potential. A linear current-voltage relationship (IV curve – lower panel) was obtained from a cell attached patch containing spontaneously active CFTR. Channel conductance was ~8.0 pS.

Basal activity of channel current was recorded from cell attached patches in MNSE cells to establish a measurement of unstimulated CFTR function. In the representative recording shown, two discernable channels were observed. Channel conductance was ~8 pS, consistent with CFTR (Figure 3).

### Resveratrol enhances CFTR channel activity

**Figure 4:**
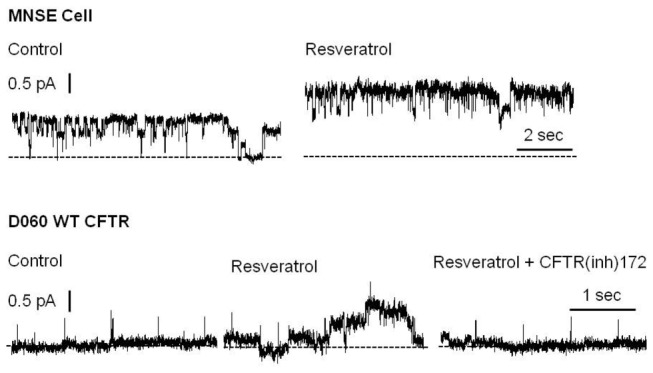
Resveratrol stimulates single CFTR channel activity. Under non-phosphorylating conditions, application of resveratrol enhanced channel activity in both MNSE (upper tracing) and D060/HEK293 cells expressing exogenous WT CFTR (bottom tracing). Patches were excised and spontaneous channel function recorded at – Vcom = +50 mV. Activity was stimulated within seconds of application of 100 µM resveratrol. Similar enhancement was observed in doxycycline induced, CFTR-expressing D060 cells and abrogated by addition of CFTR(inh)172. Dotted lines indicate zero current level.

In the excised, inside-out patch clamp configuration, segments of the apical plasma membrane sealed within the tip of the patch pipette were evaluated following direct application of test compounds to the intracellular surface. In patches from apical membranes of MNSE cells, and in the absence of ATP and PKA, resveratrol stimulated an ~8 pS chloride channel consistent with CFTR potentiation. These experiments represent the first to characterize single channel CFTR in freshly isolated/low passage number sinonasal epithelia. Patches were excised and spontaneous activity recorded at -Vcom = +50 mV. Channel function was enhanced within seconds of application of 100 µM resveratrol (Figure 4, Upper Tracing). This observation was confirmed in D060/HEK293 cells expressing exogenous WT-CFTR (Figure 4, Lower Tracing). Under cell-free conditions, an increase in channel open probability (rather than new channel insertion) was found to account for current activation. Complete inhibition of stimulated current by CFTR(inh)-172 further supports the finding that CFTR activity is augmented by resveratrol. 

### Resveratrol increases open probability of CFTR

**Figure 5:**
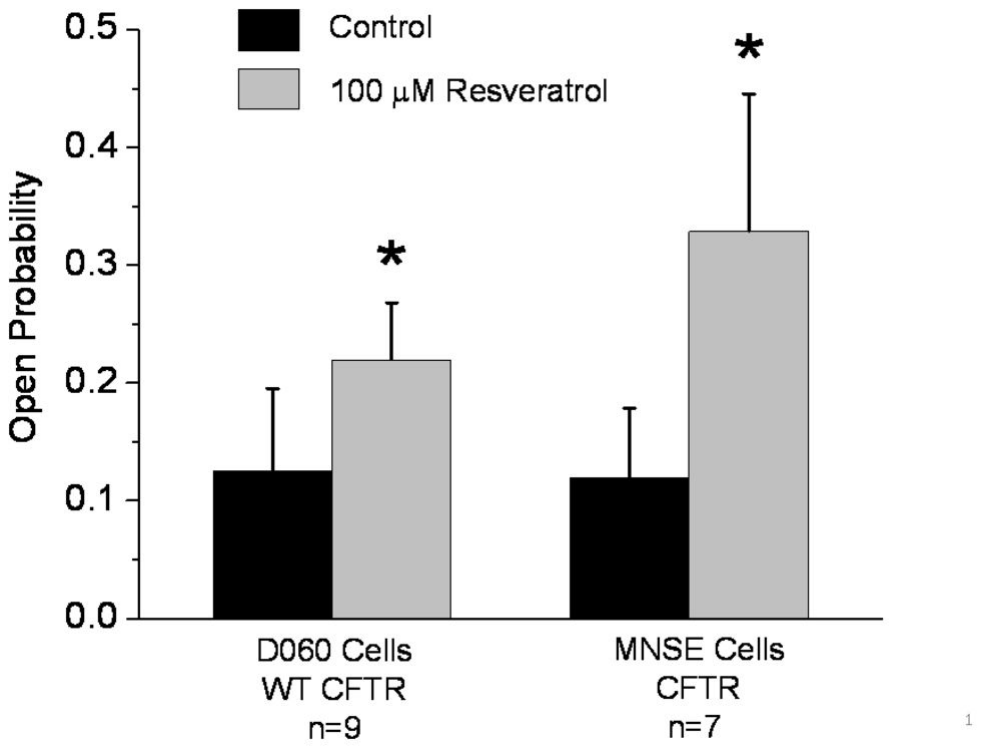
Open channel probability. Open probability (NPo/N where *N* represents channel number) was calculated from single channel recordings under control conditions and after perfusion with 100 µM resveratrol obtained in similar experiments to those displayed in Figure 4. Data are presented as mean ± SEM. Data points were compared with control using paired Student's t test (*P<0.05).

Open probability (NPo/N where *N* represents channel number) was calculated from single channel recordings under control conditions and following perfusion with 100 µM resveratrol. Open probability was increased in a statistically significant fashion by addition of resveratrol in MNSE cells (0.329 ± 0.116 vs. 0.119 ± 0.059 for control, p<0.05). This finding was confirmed in D060/HEK293 cells expressing WT-CFTR, where resveratrol significantly increased open probability compared to control (0.22 ± 0.048 vs. 0.125±0.07, p<0.05) (Figure 5). 

### Resveratrol increases ASL depth

**Figure 6:**
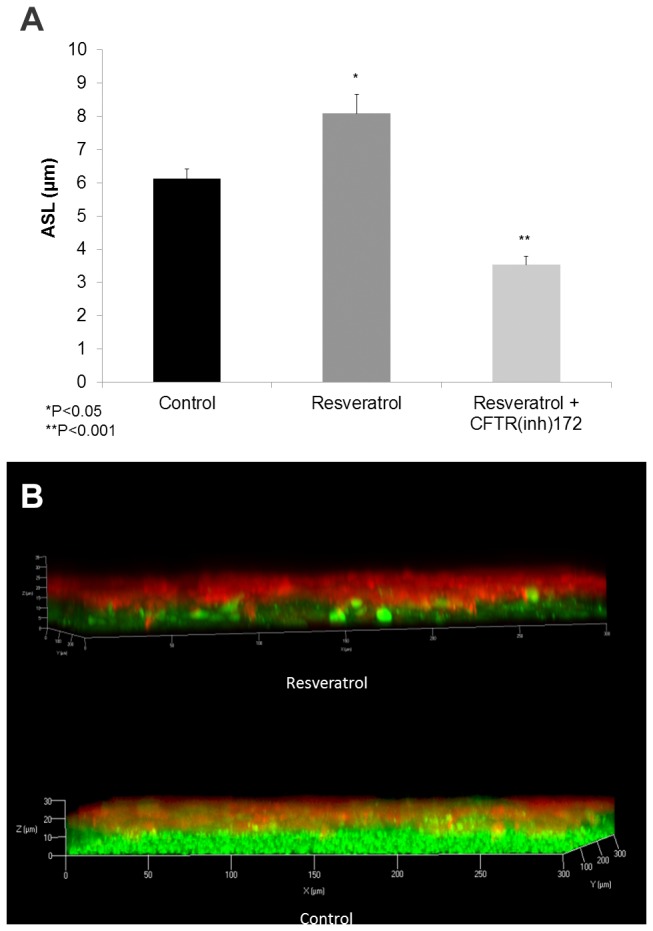
Resveratrol hydrates ASL. Resveratrol significantly increased ASL depth (in μm) in murine nasal septal epithelial cultures (8.08±1.68 vs. 6.11±0.47, control, *p<0.05). Addition of CFTR(inh)-172 inhibited resveratrol-dependent ASL accumulation (3.54±0.34, **p<0.001) (A). Confocal images demonstrate treatment effect from stimulation of apical Cl^-^ secretion and enhanced CFTR channel Po (Red-ASL; Green-Cell marker) (B).

Resveratrol increased ASL depth in MNSE cultures (in μm: 8.08±1.68 vs. 6.11±0.47, DMSO control, *p<0.05, n ≥ 5 per condition) (Figure 6) indicating a robust treatment effect resulting from stimulation of apical Cl^-^ secretion and enhanced CFTR channel Po. ASL hydration was significantly diminished by addition of the specific CFTR inhibitor CFTR(inh)-172 (3.54±0.34, **p<0.001). CFTR(inh)-172 also suppresses constitutively activated CFTR, accounting for the overall decrease in ASL depth.

### Resveratrol does not increase CFTR mRNA

**Figure 7:**
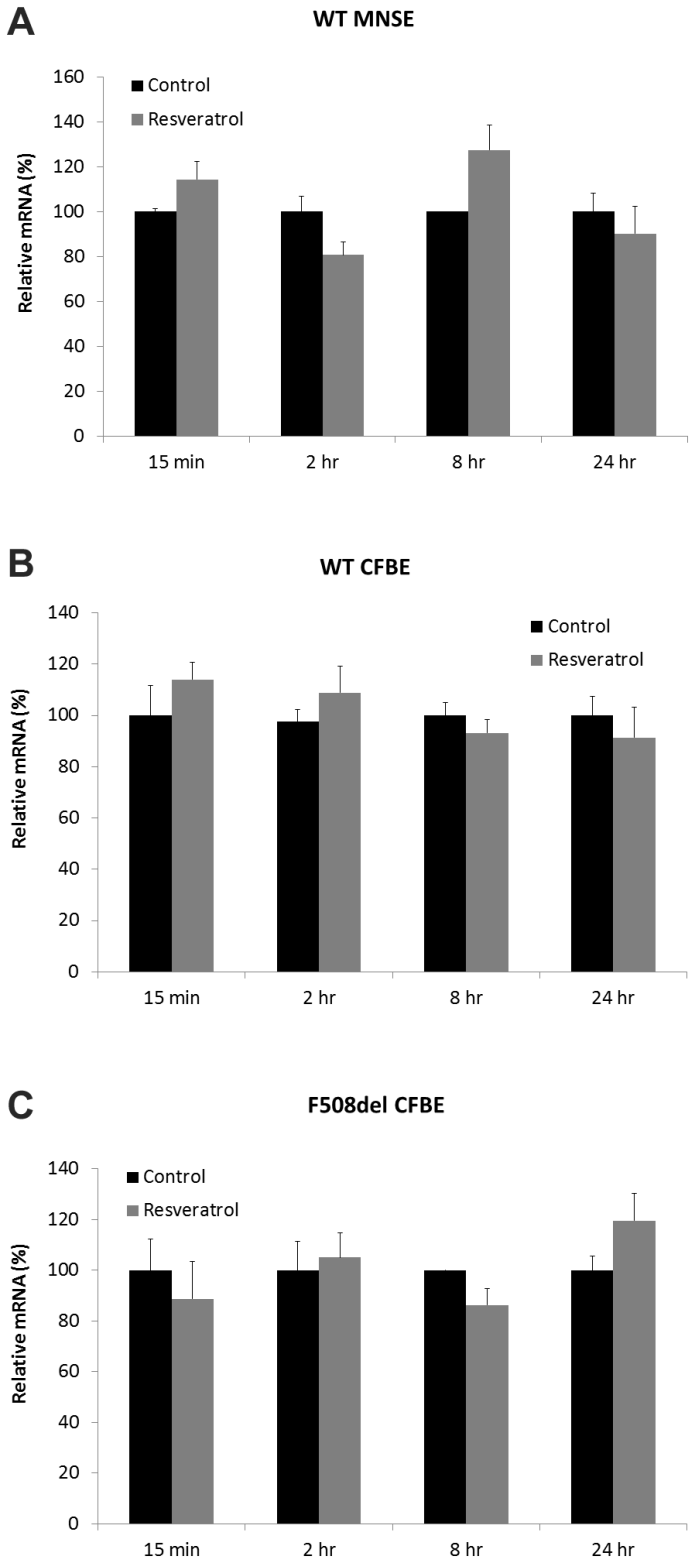
Resveratrol does not increase CFTR mRNA. Quantitative RT-PCR reveals no relative increase in CFTR mRNA following 15 minute, 2 hour, 8 hour, or 24 hour incubation with resveratrol compared to DMSO control in primary MNSE, WT CFBE, or F508del CFBE cells.

Previous findings in certain recombinant cell lines have suggested that resveratrol may increase steady state CFTR mRNA[25]. MNSE and CFBE (both WT and F508del) cultures were incubated with resveratrol for up to 24 hours and no changes in relative mRNA expression were observed (Figure 7), indicating the ASL effects noted here are not attributable to elevated CFTR expression. 

## Discussion

Very recently, CFTR activation has been shown to confer robust therapeutic benefit in CF, a disease characterized by defective MCC. The compound tested in these earlier studies (VX-770, ivacaftor) has now been FDA approved for use in a subset of individuals with CF, but is very expensive (approximately $300,000/year in the U.S.). Moreover, the mechanism that underlies activity of the drug is not known, and long term safety remains under evaluation[6]. Resveratrol is safe, as active as ivacaftor with regard to wild type (and temperature corrected F508del) human CFTR stimulation, and has additional beneficial characteristics that include well established usefulness as an anti-inflammatory. Resveratrol exhibits very strong potentiation of CFTR ion transport following topical administration as shown here. Conservation of robust CFTR potentiation across several mammalian species provides a means to study MCC activation in animal models (unlike ivacaftor, which has no effect on murine or porcine CFTR in primary nasal airway epithelium (data not shown))[26]. Just like ivacaftor, future strategies employed to potentiate F508del CFTR would require the co-administration of a “corrector” molecule (e.g. lumacaftor, Vertex pharmaceuticals, Cambridge, Ma).

Flavonoids represent a group of small, plant based molecules that have engendered scientific interest due to their possible health benefits. Resveratrol lacks the central ring of a three ring flavone backbone, but is structurally quite similar to members of the flavonoid family. The compound is present at high levels in peanuts, some flowering plants and red fruit[10,27]. Anti-inflammatory and antioxidative properties of resveratrol[27,28] have been attributed to inhibition of the transcription factor NF-κB and activator protein-1(AP-1)[11]. Earlier findings from our laboratory showed potent anti-inflammatory properties of the drug in primary airway epithelial cell cultures and indicated that resveratrol could also stimulate Cl^-^ secretion[12]. 

The importance of a new compound capable of activating fluid and electrolyte secretion and mucus clearance in sinonasal epithelium is evident, but the molecular mechanism underlying this activity (e.g. direct effects on CFTR vs. modulation of basolateral epithelial K^+^ channels, cellular cotransporters that establish the electrochemical driving force for Cl^-^ secretion, signaling mechanisms, etc.) have not been established previously. The present findings strongly indicate that resveratrol activates CFTR and enhances ASL depth. The drug was shown by single channel patch clamp analysis to enhance CFTR by increasing channel open probability in both wild type MNSE cells and a recombinant CFTR expressing cell line (D060). Direct effects on the channel are inferred because the cAMP/PKA signaling components are absent in the excised, inside out patch clamp technique. Since acquired defects of CFTR in chronic diseases such as sinusitis can result in both decreased CFTR channel expression and increased turnover in nasal airway epithelium[29,30], potentiating CFTR channels already situated in the cell membrane could serve as an effective strategy for reversing ASL dehydration among afflicted individuals with CRS.

 ASL depth was examined by confocal laser scanning microscopy following exposure to resveratrol, and increase in hydration attributable to potentiating effects on CFTR channels was confirmed. This activity was blocked by CFTR(inh)-172. CFTR channel potentiation in the excised, inside-out patch configuration effectively excludes an alternative cellular pathway (outwardly rectifying channels by resveratrol). CFTR activation that is independent of protein kinase A provides additional evidence of direct binding of the compound to CFTR and represents an optimal approach to directly stimulate CFTR, fluid and electrolyte transport, and ASL hydration. 

## Conclusion

The data presented here provide direct evidence that resveratrol enhances ASL hydration by increasing CFTR channel open probability. Recent evidence in human subjects has established that CFTR activation can have dramatic effects on airway MCC. We therefore propose a strategy using agents such as resveratrol for treatment of CRS, a common disease of airway MCC for which new treatment options are needed. Clinical trials utilizing resveratrol as a topical therapeutic to increase mucociliary transport and ASL hydration in sinus disease are indicated.
