# Correction: Resveratrol Enhances Airway Surface Liquid Depth in Sinonasal Epithelium by Increasing Cystic Fibrosis Transmembrane Conductance Regulator Open Probability

**DOI:** 10.1371/annotation/d852ff1d-2824-4f4d-80f2-9be7a42a5f25

**Published:** 2014-01-02

**Authors:** Shaoyan Zhang, Angela C. Blount, Carmel M. McNicholas, Daniel F. Skinner, Michael Chestnut, John C. Kappes, Eric J. Sorscher, Bradford A. Woodworth

 Figure 4 was incorrectly posted as both Figure 3 and Figure 4. Please view the correct Figure 3 here: 

**Figure:**
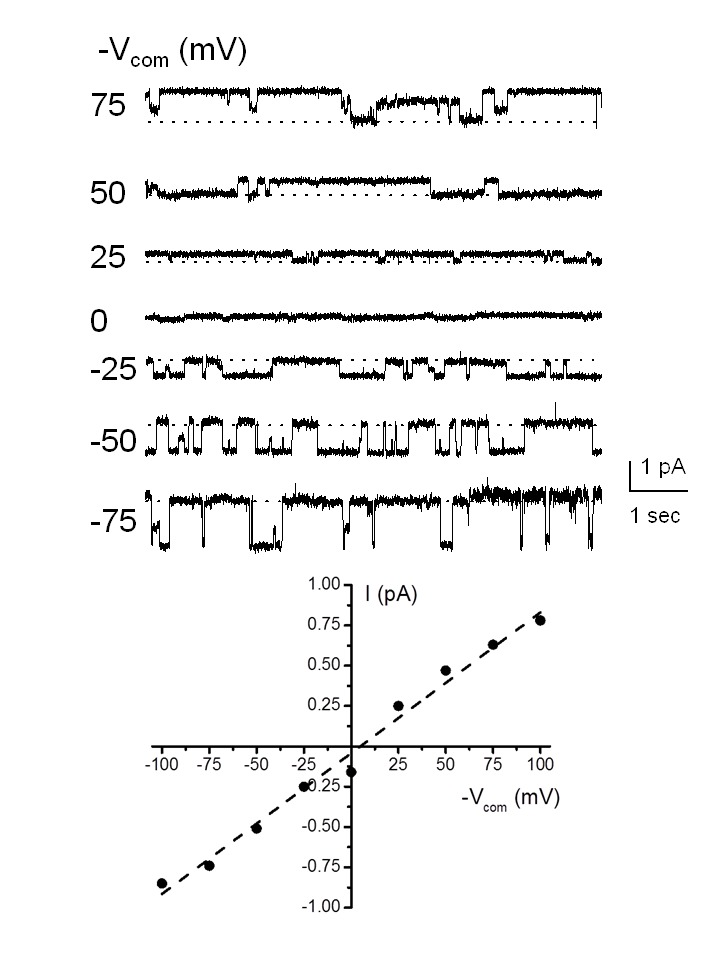


f

